# Efficient Computation of Two-Electron Reduced Density Matrices via Selected Configuration Interaction

**DOI:** 10.1021/acs.jctc.2c00738

**Published:** 2022-10-05

**Authors:** Jeremy P. Coe, Andrés Moreno Carrascosa, Mats Simmermacher, Adam Kirrander, Martin J. Paterson

**Affiliations:** † Institute of Chemical Sciences, School of Engineering and Physical Sciences, Heriot-Watt University, EdinburghEH14 4AS, U.K.; ‡ EaStCHEM, School of Chemistry, University of Edinburgh, EdinburghEH9 3FJ, U.K.

## Abstract

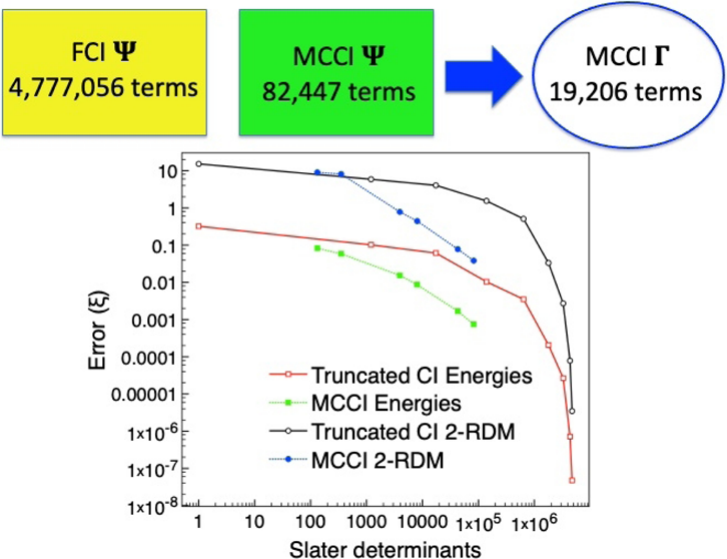

We create an approach to efficiently calculate two-electron reduced density matrices (2-RDMs) using selected configuration interaction wavefunctions. This is demonstrated using the specific example of Monte Carlo configuration interaction (MCCI). The computation of the 2-RDMs is accelerated by using ideas from fast implementations of full configuration interaction (FCI) and recent advances in implementing the Slater–Condon rules using hardware bitwise operations. This method enables a comparison of MCCI and truncated CI 2-RDMs with FCI values for a range of molecules, which includes stretched bonds and excited states. The accuracy in energies, wavefunctions, and 2-RDMs is seen to exhibit a similar behavior. We find that MCCI can reach sufficient accuracy of the 2-RDM using significantly fewer configurations than truncated CI, particularly for systems with strong multireference character.

## 1 Introduction

The molecular Hamiltonian incorporates at most two-particle interactions. Hence, the two-electron reduced density matrix (2-RDM) is all that is needed to compute the energy, as well as the expectation value of any other one- or two-electron operator. This is of particular interest for the calculation of electron scattering and X-ray scattering from molecules where the experimental observable is obtained from the Fourier transform of the 2-RDM.^1−7^ It also plays a crucial role in the efficient implementation of analytic energy gradients for configuration interaction (see, *e.g.*, ref (8)). The 2-RDM enables electron pair intracules to be calculated to study electron correlation^9,10^ and explicit-*r*_12_ correlation corrections to be included perturbatively.^11^ Furthermore, it facilitates the computation of exchange–correlation holes, which are of interest for improving the approximate functionals in density-functional theory (see, *e.g.*, ref (12)). Transition 2-RDMs are an essential part when calculating analytic non-adiabatic coupling matrix elements,^13^ and permit natural transition geminals to be found for the qualitative characterization of doubly excited transitions.^14^ Two-electron operators also occur in approximations to relativistic quantum chemistry^15^ via the Breit-Pauli Hamiltonian and allow spin–orbit coupling calculations to go beyond one-electron operators with effective nuclear charges. For a single-particle basis set of size *M*, the 2-RDM cannot have more that *M*^4^ terms, in contrast to the combinatorial scaling of the full configuration interaction (FCI) wavefunction. With respect to calculating the expectation values of one- and two-electron operators, one may therefore regard the 2-RDM as a lossless compression of the wavefunction. Using this representation can thus enable large gains in efficiency compared with the storage, transfer, and manipulation of the full wavefunction.

If we consider water, use the cc-pVDZ basis, and do not exploit symmetry, there are almost two billion Slater determinants in the FCI wavefunction, but fewer than 400,000 terms constitute the 2-RDM, amounting to just 0.018% of the size of the wavefunction. We note that the wavefunction itself has been the focus of compression, for example, by controlling the number of non-zero coefficients in an FCI algorithm,^16^ and one could also consider selected configuration interaction methods as a form of this, albeit one where there will be some trade-off between size and accuracy.

Although the FCI wavefunction is the most accurate for a given basis, considering the number of Slater determinants required, its calculation is currently possible only for small systems and basis sets. When wavefunctions have numerous important configurations, often termed multireference problems, elegant and efficient approaches based on small corrections to a single determinant, for example, CCSD^17^ or CCSD(T),^18^ can perform poorly,^19^ and multireference coupled cluster methods are still being developed. These cases, such as stretched bonds or excited states, can require the use of CASSCF^20^ to achieve qualitative results, often proceeded by MRCI^21^ or CASPT2^22^ for quantitative accuracy. However, such an approach can rapidly become computationally intractable, like FCI, as the active space becomes larger. However, small active spaces can be insufficient or require expert knowledge to construct and thus introduce the risk of bias. One promising class of methods to model multireference problems in an unbiased manner with tractable wavefunctions is the selected configuration interaction^23−32^ where a compact wavefunction is built up by selecting and removing configurations based on relevant criteria and performing multiple diagonalizations. In this work, we consider the selected CI approach of Monte Carlo configuration interaction (MCCI)^24,33,34^ where configurations are chosen randomly, and we appraise its ability to approximate the FCI 2-RDM. Therefore, we investigate two improvements in the efficiency when constructing 2-RDMs: the first is in the substantially smaller configuration space required for MCCI that can allow systems that are too large for FCI to be modeled, while the second is achieved by combining ideas from alpha and beta string FCI^35,36^ with recent advances in implementing the Slater–Condon rules using hardware bitwise operations.^37^

In this paper, we initially discuss the calculation of the 2-RDM from Slater determinant wavefunctions and ways to improve the efficiency. Next, we summarize the MCCI approach and how we can convert configuration state functions (CSFs) to Slater determinants by applying the projector operator approach of ref (38) to enable the use of the efficient 2-RDM approach for pure spin states. We calculate FCI 2-RDMs for wavefunctions of up to 2.4 billion determinants, then compare MCCI and truncated CI to the FCI values for energies, 2-RDMs, and wavefunctions for a range of molecules, which include stretched bonds and excited states.

## 2 Theory

### 2.1 Derivation of the 2-RDM Equations

In the notation of second quantization, the standard non-relativistic Hamiltonian in quantum chemistry may be written in terms of molecular spin orbitals as^39^
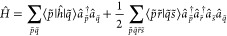
1

For molecular spatial orbitals, this becomes
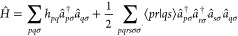
2where  creates molecular spatial orbital *p* of spin σ while  annihilates molecular orbital *s* of spin σ^′^. Here, *p*, *q*, *r*, and *s* range over all molecular spatial orbitals *M* in the basis set, and σ is the spin coordinate which is either up (↑) or down (↓). The one-electron integrals, *h*_*pq*_, are
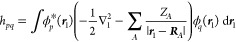
3where *Z*_*A*_ and ***R***_*A*_ are the charge and position of nucleus *A*, and ***r***_1_ is the electron coordinate. The two-electron integrals ⟨*pr*|*qs*⟩ are
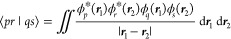
4and the expectation value  gives the energy
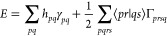
5Here, γ_*pq*_ is an element of the spin-free one-electron reduced density matrix (1-RDM) in terms of spatial molecular orbitals
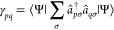
6and Γ_*prsq*_ is an element of the spin-free 2-RDM
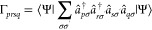
7

The factor of 1/2 in eq 5 is sometimes incorporated into the 2-RDM, but we highlight that this is not carried out in this work. As we consider the non-relativistic Hamiltonian, which does not contain spin, then the spin-free 2-RDM is sufficient to give the energy or any other spin-independent one- or two-particle property. If we wished to go beyond this and calculate spin-dependent properties, then the four combinations of spin in eq 7 could be considered separately to give spin blocks of the 2-RDM. To calculate Γ_*prsq*_ from an expansion of Slater determinants |Ψ⟩ = ∑_*i*_*c*_*i*_|Φ_*i*_⟩, we have
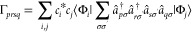
8

We initially set the 2-RDM to zero and then consider all pairs of determinants (*i* and *j*) for the following three situations of two, one, and zero differences in the lists of orbitals comprising the determinants:

- Two differences

For two differences, we have spin orbitals  as the differences in the determinant on the right where *s* is the spatial orbital of the first difference and has spin σ_*s*_. Furthermore, spin orbitals  are the differences in the determinant on the left where *r* is the spatial orbital of its first difference.

When considering all contributions to the 2-RDM and swapping operators to return to the form , we have four cases in total, and the 2-RDM is updated according to algorithm 1
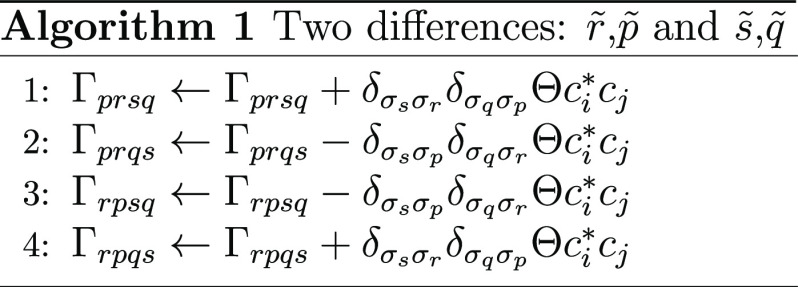


Here, Θ is the sign that results from placing the determinants in maximum coincidence and  is the Kronecker delta of the spins of  and .

- One difference

In this case, there is a single difference in the lists of spin orbitals from the two determinants, spin orbital  for the right determinant and spin orbital  for the left determinant. Looping over all other spin orbitals  in the determinant, we have algorithm 2 where again we use  for a spin orbital and *s* for the corresponding spatial orbital.
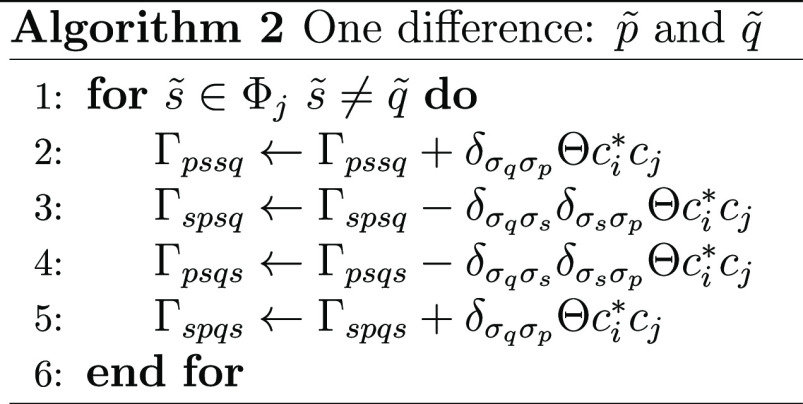

- No differences

Finally, we have no differences where all ordered pairs of spin orbitals  and  in the determinant are looped over (algorithm 3).
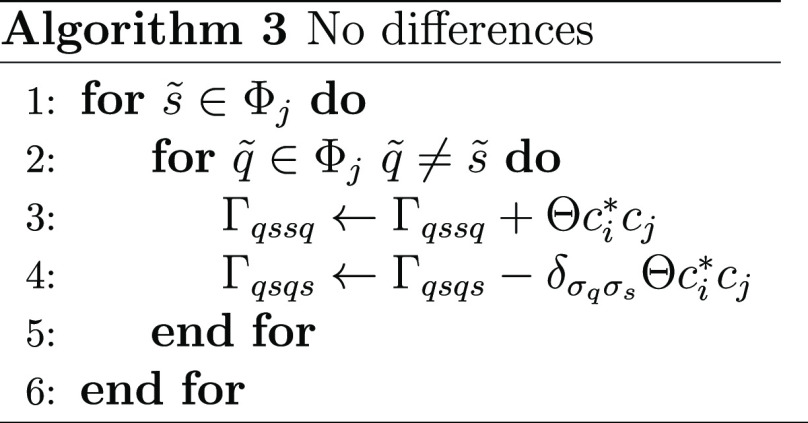


We validate the derivation of Γ_*prsq*_ by verifying that it gives the correct energy *via*eq 5. To do this we note that, from the definition of the RDMs and the anticommutation relations for the creation and annihilation operators, the 1-RDM may be calculated from the 2-RDM using
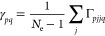
9where *N*_e_ is the number of electrons in the system. As the 2-RDM is all that is needed to give the energy, or any two-electron property, we note that there has also been impressive work on approximating the 2-RDM to do away with the wavefunction altogether,^40−42^ and recent research has advanced these approaches so that they can be used for geometry optimization.^43,44^ However, variationally optimizing the 2-RDM can lead to unphysical solutions unless further constraints are applied (see, *e.g.*, ref (45)). Our FCI 2-RDMs in this work for wavefunctions of up to 2.4 billion determinants should therefore also provide useful benchmark data for researchers developing methods based on approximate 2-RDMs.

For large FCI wavefunctions, the calculation of the 2-RDM can become onerous even if we consider only *i* ≤ *j*. However, each determinant has non-zero 2-RDM contributions only from those determinants it is linked to by a single or double substitution. Rather than checking every determinant to see whether it contributes, we only consider the singles and doubles for each determinant. This means that, when not considering symmetries, instead of  terms we have, for fixed numbers of electrons, *O*(*N*_SD_*M*^2^), where *M* is the number of basis functions. This is due to the doubles dominating as there are *N*_α_(M – *N*_α_)*N*_β_(M – *N*_β_) double substitutions of mixed spin. When the number of electrons is not fixed but much smaller than the number of basis functions, the scaling will therefore be . As the number of determinants increases combinatorially with basis size for FCI, this will lead to a substantial reduction in the number of terms. Of course, if calculating the singles and doubles is computationally expensive, we will not have accelerated the calculation of the 2-RDM. Hence, to do this efficiently, we use ideas from fast implementations of FCI, which we discuss next.

### 2.2 Alpha Beta String Efficient FCI Approach for 2-RDM Construction

Generally, for FCI implementations, the main computational cost is applying the Hamiltonian matrix to trial vectors, ***Hb*** = ***d*** (see, *e.g.*, ref (36)), in the Davidson algorithm^46^ for iterative diagonalization. For large FCI, storing ***H*** requires too much memory, so its elements are calculated on the fly. For ∑_*k*_*H*_*ik*_*b*_*k*_, only terms which have a determinant Φ_*k*_ formed by a single or double substitution from determinant Φ_*i*_ need to be considered. Early in the development of FCI calculations it was noted^35^ that, for , the locations of singles and doubles was only needed for the lists of α orbitals specifying determinants (α strings), thereby considering only  rather than *N*_FCI_ terms. Furthermore, the matrix elements for double α substitutions are independent of the β and vice-versa, so these matrix elements can be precomputed.

Based on this approach, we implement an efficient parallel FCI program. We briefly sketch the procedure below when symmetry is not used and *M*_s_ = 0 so that the β are the same as the α strings. We note that the program can also work with symmetry and for *M*_s_ ≠ 0. In the latter case, we need to store the locations of the singles and doubles for the β strings as well.

We generate all allowed α strings, and for each one, we store the location of its single and double excitation strings. We also store its double excitation matrix elements and the single excitation orbitals together with the sign from putting the single substituted string in maximum coincidence with the original. The diagonal part of the Hamiltonian matrix is also stored. For the later 2-RDM calculation, we also retain the double excitation orbitals and the signs for the doubles.

We map the combined α string location (α_loc_) and β string location (β_loc_) to a FCI vector location (FCI_loc_) using
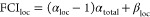
10where α_total_ is the total number of α strings.

For the calculation of Hamiltonian matrix elements and the signs from putting strings in maximum coincidence, we use the approach of ref (37) to exploit hardware bit operations in modern CPUs for efficiency. This depends on using the hardware instructions of *popcnt*, to count the number of ones (orbitals) in the binary representation of an integer, and *trailz*, to give the number of trailing zeroes. For example, we get the number of orbitals of a given irrep in an α string by calculating its bitwise AND with a symmetry bitmask (an integer whose binary representation only has ones for all the orbitals of that irrep) followed by using *popcnt*. The list of orbitals from an α string can be found by using *trailz* to give the lowest numbered occupied orbital and then taking the bitwise AND of the α string subtract one with the original α string to give a new α string and repeating until this is zero. For *d*_*i*_ = ∑_*k*_*H*_*ik*_*b*_*k*_, different *i* are considered in parallel by using OpenMP to parallelize the α string loop. If the FCI vector is sufficiently small, we keep all of the ***b*** and ***d*** vectors in memory. Otherwise, we keep all but three of these vectors on disk.

When the Davidson algorithm has converged, the FCI vector and structure are used to calculate the 2-RDM efficiently by using the FCI α string approach to consider only the singles and doubles for each determinant. This step is not parallelized as there would be multiple processes simultaneously trying to modify the same data (race conditions) due to different determinants contributing to the same 2-RDM elements.

We note that the standard *N*-resolution method for direct FCI will scale as *O*(*N*_SD_*M*^4^) and the minimal operation-count method scales as  when the number of orbitals is larger than the number of electrons.^47^ In ref (47) Helgaker, Jorgensen, and Olsen provide an approach for the calculation of the 1-RDM but not the 2-RDM where they note that “a well-designed algorithm for the construction of the two-electron density matrix will have an operation count identical to the count of a direct CI iteration for a two-electron operator.” Hence the present method, although the prefactor may be different, would be expected to scale similar to a well-designed approach for calculating the 2-RDM based around the minimal operation-count method.

### 2.3 Efficient Method for General Slater Determinant Wavefunctions

To efficiently compute 2-RDMs in the case of a general Slater determinant wavefunction, we make use of the same methods presented above adapted to a subset of the configurational space. As stated before, Slater–Condon rules serve to discriminate the non-zero overlaps between the different Slater determinants and, making use of the bitwise operations from ref (37), one can apply the methods presented for FCI to a general wavefunction. We have developed an efficient search algorithm that calculates the single and double excitations for each of the Slater determinants considered and searches for them in the configuration interaction vector expansion. Considering that not all possible determinants are present in a general case, the speed-up for a general wavefunction is less than that for FCI, and the necessity for a search algorithm also decreases this compared to a calculation based solely on the approaches discussed in the previous sections.

### 2.4 Truncated CI and MCCI

If the FCI vector is too large to be stored in memory, one straightforward approximation is to limit the configuration space to determinants formed by single and double substitutions from the reference (CISD). This can be systematically improved to include triple substitutions (CISDT), then quadruple substitutions (CISDTQ), and so forth. In this work, we denote CISDTQ as CI(4), CISDTQQ as CI(5), and so on.

If the FCI wavefunction is well-described by small corrections to a single reference, one would expect CISD to be reasonably accurate. However, when there are many important configurations, much higher levels of substitutions may be needed for accurate results. For these multireference situations, the calculation of a sufficiently accurate truncated CI wavefunction can rapidly become unwieldy as we are compelled to include higher-level substitutions. However, in quantum chemistry, many of the determinants often have negligible importance in the wavefunction.

Selected configuration interaction approaches seek to exploit this observation and find compact wavefunctions that can describe multireference problems sufficiently well. To do this, they build up a wavefunction by repeatedly adding and removing configurations based on the results of diagonalizing the Hamiltonian matrix in the current space.

One approach that has been demonstrated to be effective is MCCI.^24,33,34^ This removes any possible bias by adding new configurations randomly and then removing newly added configurations if their absolute coefficient in the resulting wavefunction is less than the cutoff value *c*_min_. Every 10 iterations, all configurations are considered for deletion, and the calculation ends when the energy converges to within a threshold. In contrast to efficient methods for FCI, the Hamiltonian matrix is stored in memory in MCCI as the configuration space is much smaller, and the Hamiltonian matrix is not expected to change drastically from one iteration to the next. This saves recalculating all the matrix elements on every iteration. In addition, the wavefunction coefficients for each state of interest are used as initial ***b*** values for the next iteration to accelerate the convergence of the Davidson diagonalization. We use MOLPRO^48^ to provide the one-electron and two-electron molecular orbital integrals for MCCI.

In the original MCCI program,^34^ the representation used for a determinant is orbital ordering then spin, for example, ϕ_1α_ϕ_1β_, ϕ_2α_ϕ_3β_ rather than spin then orbital ordering, for example, ϕ_1α_ϕ_2α_, ϕ_1β_ϕ_3β_, which is used for the FCI program in this work together with the efficient Slater–Condon routines of ref (37) and the efficient 2-RDM calculation. MCCI can use Slater determinants or CSFs, with the latter guaranteeing pure spin states and resulting in a smaller Hamiltonian matrix. However, the construction of the Hamiltonian matrix is much more complicated when using CSFs.^49^

### 2.5 CSFs to SDs

By using the projector approach of ref (38), we can convert CSFs to Slater determinants and then apply the efficient 2-RDM calculation. To project out wavefunctions with spin quantum number *k*, we apply the operator of ref (38)
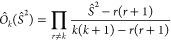
11where the product is over all possible spin quantum numbers *r* except the spin quantum number *k* we want. Here
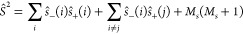
12where the operator  acts on the *i*’th spin to flip β and annihilate α, while this is reversed for  (see, *e.g.*, ref (50)).

For each list of orbitals defining a CSF, we apply the product in eq 11 from *r* = 0 to half the number of unpaired electrons, while we bypass *r* = *k*. Duplicates are removed in the small set of Slater determinants for this CSF after each application to ensure that we do not waste time acting on the same determinant twice. The determinants are then stored, and we move on to the next CSF. After all CSFs have been considered, we remove duplicates in the full set of Slater determinants. Thereby, we make the calculation faster than checking the full set for duplicates after each CSF but at the cost of more memory being required.

We verify that the procedure has worked correctly by checking that the Slater determinant expansion is normalized and that the energy and spin take the expected values. We note that recently created CSF to SD transformations for large numbers of unpaired electrons^51^ have allowed fast calculations of SD expansions from tens of millions of CSFs for low-spin states with many unpaired electrons. Furthermore, there is recent work on getting the best of both worlds through transformations that allow a hybrid CSF/SD approach for configuration interaction.^52^

## 3 Results and Discussion

We first demonstrate the speed-up in the calculation of the 2-RDM for the FCI wavefunction of the ground state of neon using the 6-31G* basis set with one frozen orbital. We then compare errors of truncated CI and MCCI with FCI for energies, 2-RDMs, and wavefunctions. Next, we consider these errors for an excited state of neon and then for CO in its equilibrium and stretched geometries, where we look at the speed-up for the 2-RDM calculation using a general Slater determinant wavefunction. Increasing the basis set size is also considered for equilibrium CO where the size/accuracy trade-off of the wavefunction for SDs *versus* CSFs is compared as well. This is also considered for the final system we look at, the *M*_s_ = 1 triplet state of O_2_.

### 3.1 Neon

Initially, we look at the ground state of neon using the 6-31G* basis set with one frozen orbital. The FCI wavefunction has 125,861 SDs here, and we quantify the multireference character for the given basis and molecular orbitals using
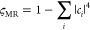
13which increases from 0 to 1 as the amount and spread of important configurations grow.^53,54^ For this basis set, the neon atom has a very low multireference character of just ς_MR_ = 0.055.

We first demonstrate the improvements in the calculation time of the 2-RDM by comparing four approaches:

1. orbital then spin ordering,
2. spin then orbital ordering and using the hardware bitwise operations of ref (37),
3. spin then orbital ordering, using hardware bitwise operations of ref (37) and only calculating *i* ≤ *j* from the wavefunction expansion,
4. spin then orbital ordering, using hardware bitwise operations of ref (37) and exploiting the FCI structure.

The timings on one thread of a 3.8 GHz 8-Core Intel Core i7 CPU are displayed in Table 1 where we see that we can get just over a ×4 speed-up compared with the initial approach when using the method of hardware bitwise operations.^37^ Coupling this with using the FCI structure makes the calculation around 220 times faster, thereby allowing us to tractably compute 2-RDMs for much larger wavefunctions. We note that the FCI structure needs to be calculated, but the FCI calculation would be carried out anyway to obtain the wavefunction and only required around 1 s using 16 threads.

**Table 1:** Timings for the Calculation of the 2-RDM from the Neon FCI Wavefunction with 125,861 SDs for Our Four Approaches as Detailed in the Text

method	time for 2-RDM (s)
1	84.1
2	20.0
3	15.7
4	0.376

We quantify the accuracies compared with FCI using the error
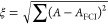
14where the sum runs over all elements in the quantity of interest *A*, which is either the energy, 2-RDM, or wavefunction coefficients. The latter have their global phases chosen to minimize the error. The errors are not normalized, so we cannot necessarily say that one quantity is captured more accurately than another for a single method, but we can compare between methods to see whether a higher level of excitation is needed in truncated CI to achieve an MCCI wavefunction error than to reach an MCCI energy accuracy.

**Figure 1:**
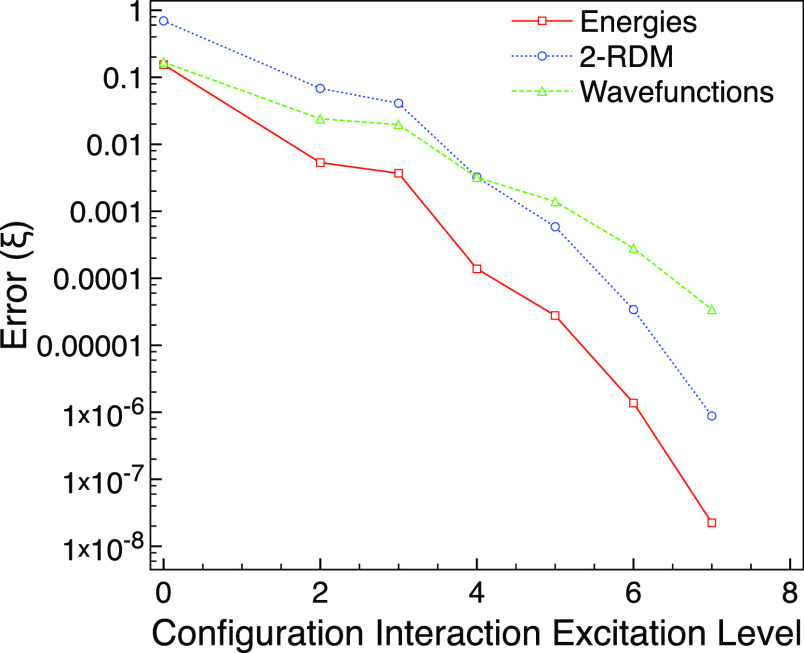
Truncated CI errors compared with FCI for the ground state of neon using the 6-31G* basis set and one frozen orbital.

We see in Figure 1 that CISD has already lowered the energy error to around 0.005 hartree and that the energy, 2-RDM, and wavefunction errors generally behave similarly on increasing the excitation level. The 2-RDM and wavefunction errors have crossed over by CI(5). However, the errors are getting quite small by this point.

**Figure 2:**
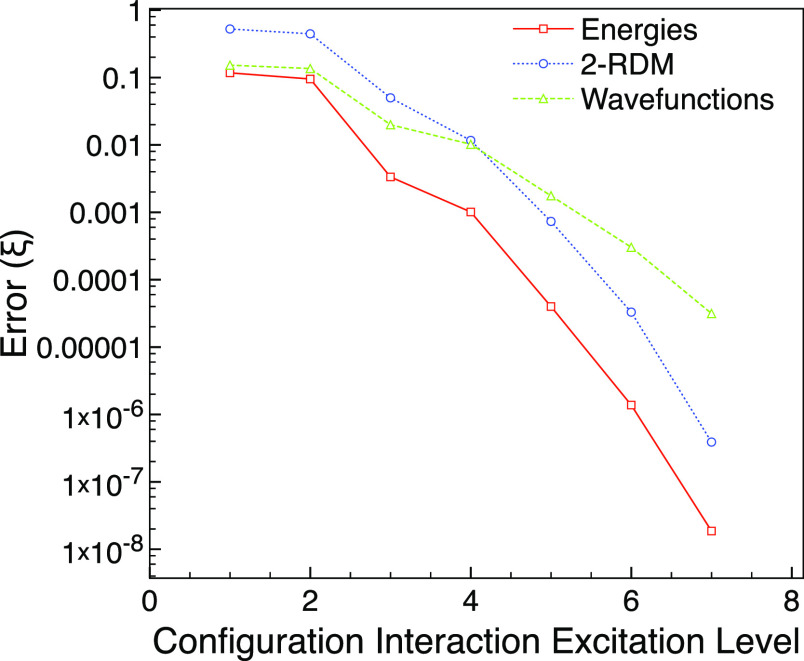
Truncated CI errors compared with FCI for the first excited A_g_ triplet state of neon with *M*_s_ = 0 using the 6-31G* basis set and one frozen orbital.

The first excited A_g_ triplet state of neon with *M*_s_ = 0 has a strong FCI multireference character of ς_MR_ = 0.848 when using the 6-31G* basis. Figure 2 shows that CISD does not improve much over CIS and neither gets sufficiently close to FCI in comparison with CISD for the ground state. For this, we need to go to CISDT to get errors that are similar to CISD in the ground-state calculation.

**Figure 3:**
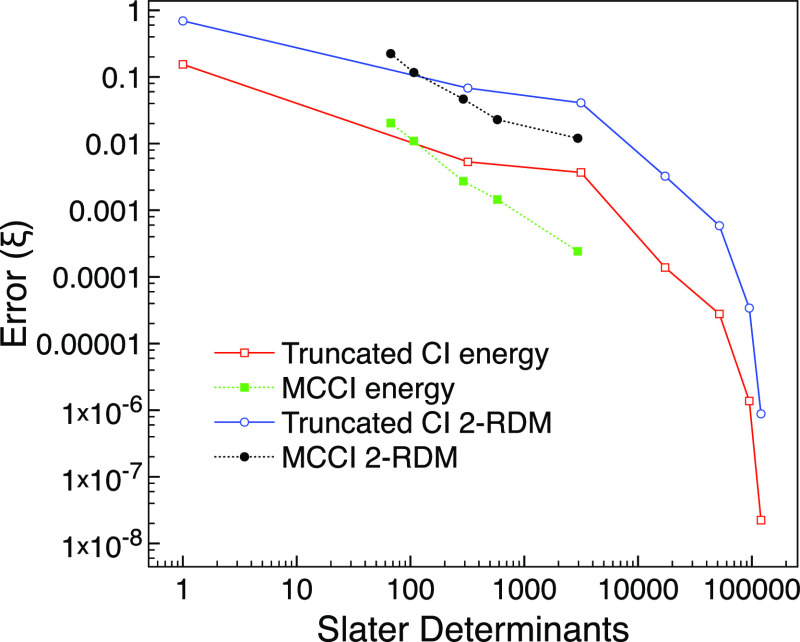
MCCI and truncated CI errors compared with FCI for the ground state of neon using the 6-31G* basis set and one frozen orbital.

Next, we consider the MCCI approach with cutoffs ranging from 0.01 to 10^–4^ and see in Figure 3 that for the ground-state of neon, MCCI can use fewer determinants than CISD, yet give a lower error than CISDT for the energy and 2-RDM.

**Figure 4:**
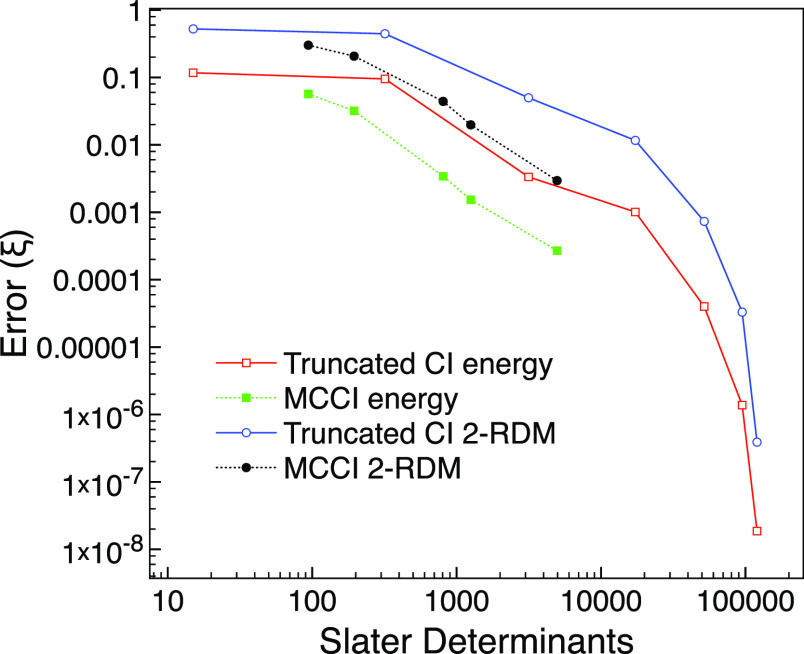
MCCI and truncated CI errors compared with FCI for the first excited A_g_ triplet state of neon with *M*_s_ = 0 using the 6-31G* basis set and one frozen orbital.

We see in Figure 4 that for the excited state, the error-to-SD ratio is noticeably lowered with MCCI. All MCCI results now have lower errors than CISD, and the plot suggests that we can achieve errors lower than CI(4) using fewer determinants than CISDT. Hence, for this problem with strong multireference character, the performance of MCCI relative to truncated CI is enhanced compared with the ground state, which had very little multireference character. This fits in with the selected CI approach being more able to find the important configurations. However, if the system is well-described by a single reference, the advantage of MCCI will be diminished as a large amount of configurations with small coefficients can only make minor improvements to the already quite accurate wavefunction.

### 3.2 CO

For CO, the 6-31G basis set, and two frozen orbitals, the FCI wavefunction consists of 4,777,056 SDs. We first consider the equilibrium bond length^55^ of 2.1316 *a*_0_ and find that ς_MR_ = 0.188, suggesting a small amount of multireference character.

**Figure 5:**
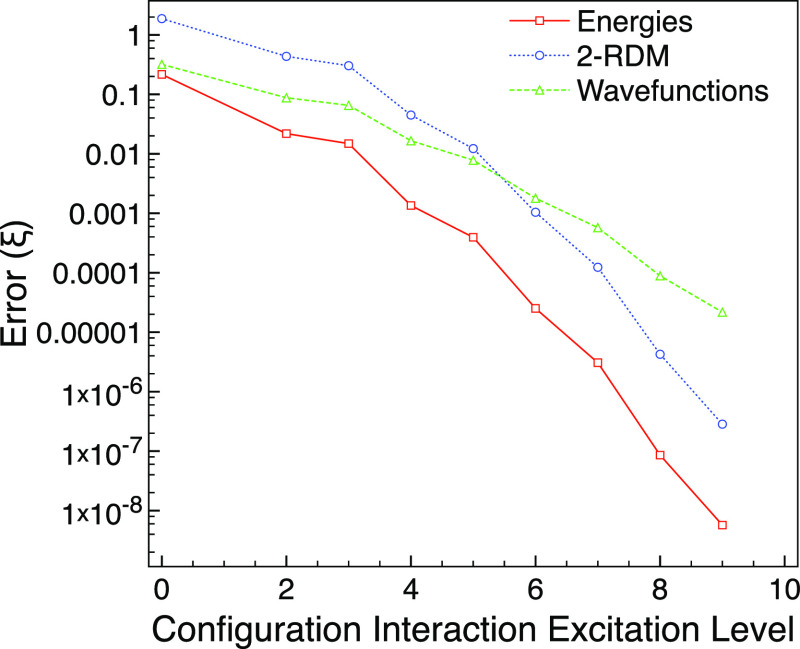
Truncated CI errors compared with FCI for the ground state of carbon monoxide using the 6-31G basis set and two frozen orbitals for the equilibrium bond length of 2.1316 *a*_0_.

As for neon, the errors follow a similar trend (Figure 5) with a crossing between the wavefunction and 2-RDM errors by an excitation level of 6 for this system.

**Figure 6:**
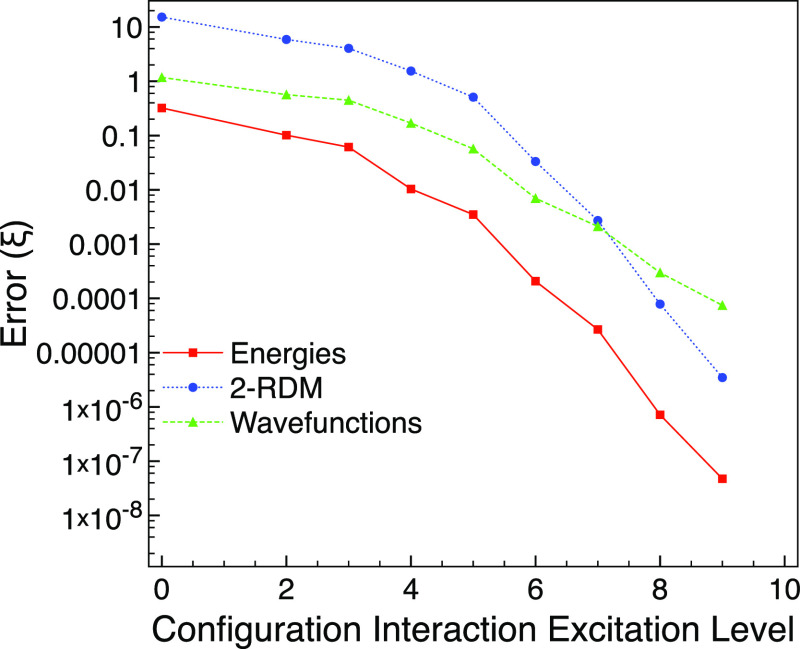
Truncated CI errors compared with FCI for the ground state of carbon monoxide using the 6-31G basis set and two frozen orbitals for a stretched bond length of 4 *a*_0_.

We also consider a stretched bond length of 4 *a*_0_, which has a very high multireference character of ς_MR_ = 0.9554. As the MCCI wavefunctions are larger than they were for neon, this system provides a good test for the improvements in the computational time required to construct the 2-RDM for a general Slater determinant wavefunction. On one thread of a 2.9 GHz Intel Xeon CPU, we find that the time to get the 2-RDM from the MCCI wavefunction with cutoff 5 × 10^–5^ is reduced by around a factor of 26 using the efficient approach of this paper. In Figure 6, we plot the truncated CI errors. We see that the errors are noticeably higher for the stretched than that for the equilibrium geometry, with the 2-RDM errors being around an order of magnitude larger. This illustrates the challenge of modeling systems with strong multireference character using highly truncated wavefunctions. If we wish to lower the error in the stretched calculation to a similar size as for CISD in the equilibrium geometry, we see in Figure 6 that CI(4) is needed for the energies but CI(5) for the 2-RDM and wavefunction. This is consistent with the expectation that it is more challenging to accurately compute the 2-RDM, which can give any one- or two-electron property, than the energy, for which the wavefunction is variationally optimized.

**Figure 7:**
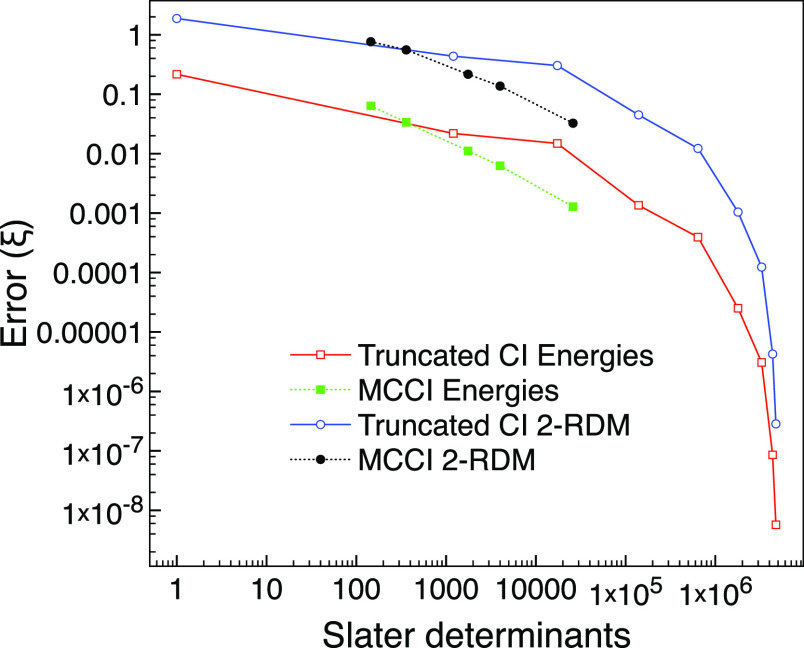
MCCI and truncated CI errors compared with FCI for the ground state of carbon monoxide using the 6-31G basis set and two frozen orbitals at the equilibrium geometry of 2.1316 *a*_0_.

Figure 7 shows that for the equilibrium geometry, MCCI with the lowest cutoff of 10^–4^ can achieve a low error comparable to CI(4) but uses a similar magnitude of determinants as CISDT. In this respect, MCCI performs slightly better than that for ground-state neon, which fits in with the multireference character being higher for carbon monoxide.

**Figure 8:**
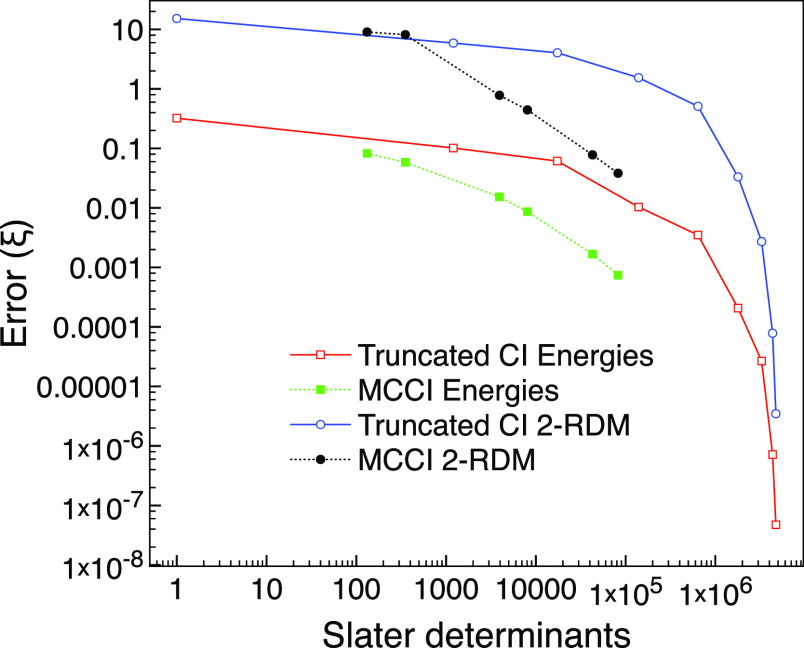
MCCI and truncated CI errors compared with FCI for the ground state of carbon monoxide using the 6-31G basis set and two frozen orbitals at a stretched geometry of 4 *a*_0_.

Figure 8 reveals that MCCI can get a 2-RDM error comparable to CI(6) for the stretched system but uses fewer determinants than CI(4). Here, CI(6) required 1,788,324 determinants, while the largest amount needed for MCCI was 82,447. This, again, demonstrates the benefit of selected CI wavefunctions when the multireference character is very strong.

Next, we consider a larger basis for CO in its equilibrium geometry. When using cc-pVDZ with two frozen orbitals, we calculate the ground-state FCI wavefunction, which has 2,414,950,976 determinants, and use it to compute the 2-RDM. Due to the size of the wavefunction, we do not retain it after the calculation, and therefore, wavefunction errors are not considered.

**Figure 9:**
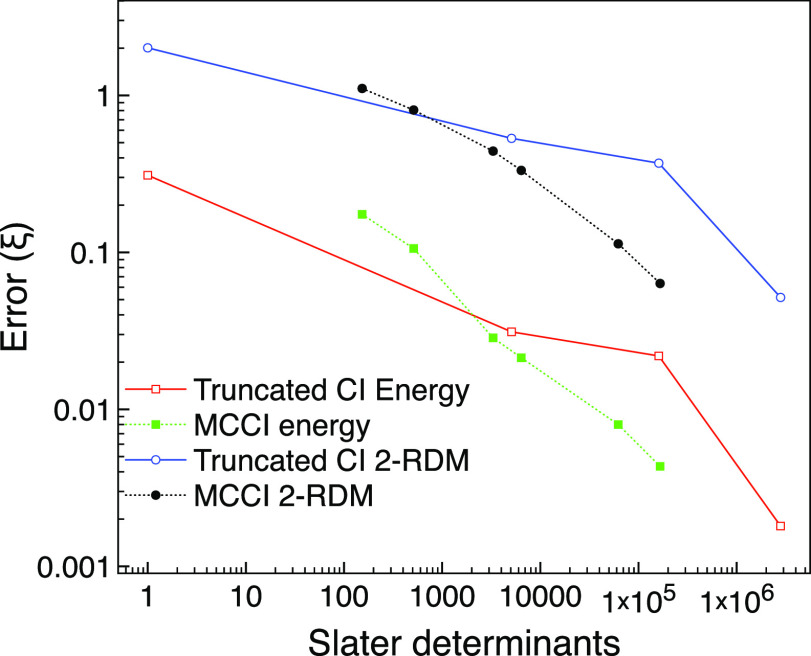
Truncated CI and MCCI errors compared with FCI against the number of determinants for the ground state of carbon monoxide using the cc-pVDZ basis set and two frozen orbitals.

The minimum MCCI cutoff is now lowered to 5 × 10^–5^, and for this wavefunction, the multireference character is found to be ς_MR_ = 0.21. We see in Figure 9 that, although MCCI does much better than CISDT for a similar number of determinants, we cannot reach CI(4) accuracy now that the FCI space is very large even with this smaller cutoff. We contrast this with the results for the smaller basis in Figure 7 where an accuracy slightly higher than that for CI(4) could be achieved by MCCI with a cutoff of 10^–4^. It is worth noting that the CI(4) calculation for cc-pVDZ has become large with 2.8 million determinants, which, together with not much multireference character, suggests that the better performance of CI(4) should be expected here.

**Figure 10:**
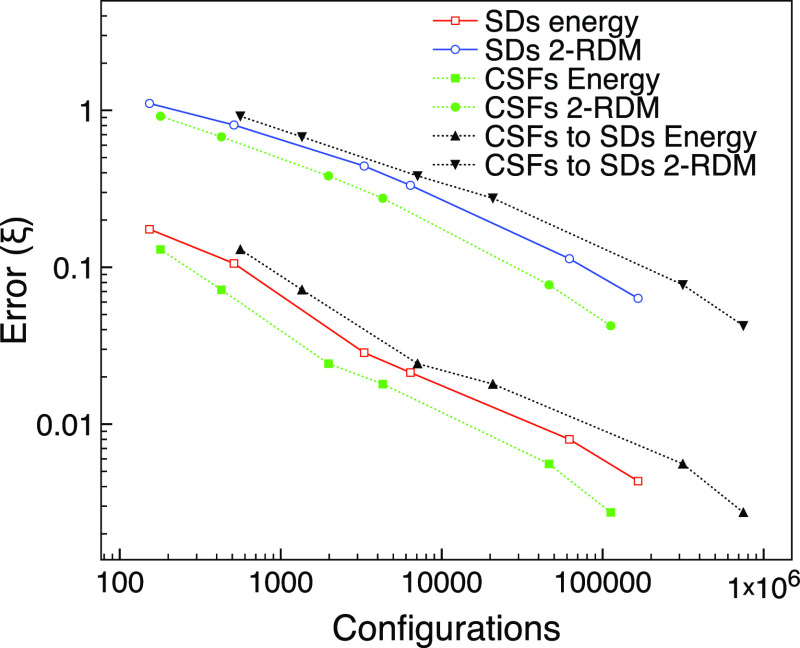
MCCI errors using SDs or CSFs compared with FCI against number of configurations for the ground state of carbon monoxide using the cc-pVDZ basis set and two frozen orbitals.

We also compare the use of CSFs with SDs. Figure 10 shows that fewer configurations are needed for a given error when using CSFs rather than SDs. However, CSFs are not necessarily improving the trade-off between size and accuracy in terms of SDs. If we convert the CSFs to SDs, we see that the corresponding number of SDs is greater for the CSF calculation than that for the SD calculation, and in Figure 10, the converted CSF error curve against configurations is above the SD error curve. Table 2 shows that the spin contamination is not high in the Slater determinant MCCI wavefunction, suggesting that the main benefit is a more compact expansion in the CSF representation for this singlet system.

**Table 2:** MCCI SD Spin Error for CO Quantified as the Difference to the Pure Singlet Total Spin Squared Expectation Value of *S*(*S* + 1) = 0

cutoff	spin error
0.01	2.2 × 10^–2^
0.005	2.3 × 10^–2^
0.001	1.5 × 10^–3^
0.0005	1.1 × 10^–3^
0.0001	8.3 × 10^–4^

### 3.3 Triplet O_2_

We finally model a system with unequal numbers of α and β electrons by considering the B_1g_ triplet state of O_2_ with *M*_s_ = 1 at the experimental bond length^56^ of 1.2075 Å. We use the 6-31G basis set and two frozen orbitals. The FCI wavefunction contains 6,248,880 SDs in this case.

**Figure 11:**
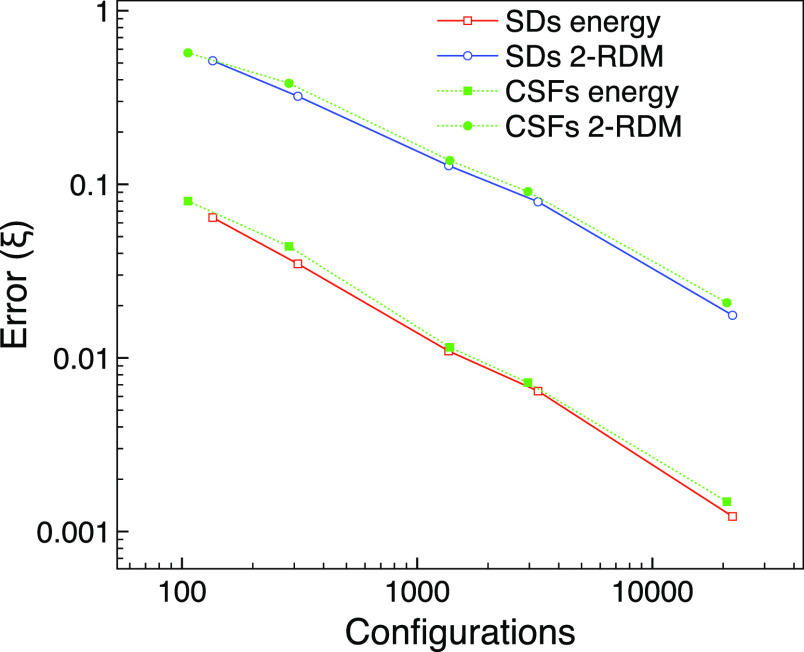
MCCI errors using SDs or CSFs compared with FCI against number of configurations for the B_1g_ ground state of oxygen with *M*_s_ = 1 using the 6-31G basis set and two frozen orbitals.

In Figure 11, we compare the errors when using SDs with CSFs for the MCCI calculation with cutoffs from 10^–2^ to 10^–4^. The multireference character for the MCCI SD wavefunction at the lowest cutoff considered was not strong at ς_MR_ = 0.178. For this system and basis, we see that CSFs give a similar error that is perhaps slightly larger for a similar number of configurations than that for SDs. This is interesting given that expanding the CSFs to SDs increases the number of configurations by around four or five times here.

The good performance of SDs for the energy might be expected to come at the cost of a large deviation from pure spin states. However, Table 3 reveals that the spin contamination is small for SDs here and decreases with the number of configurations. Therefore, in terms of SDs, the wavefunctions are much more compact than that in the CSF calculation with only a small amount of spin contamination as a penalty. The difference from pure spin is slightly less than that for CO (Table 2) for a given cutoff, but the order of magnitude is similar.

**Table 3:** MCCI SD Spin Error for O_2_ Quantified as the Difference to the Pure Triplet Total Spin Squared Expectation Value of *S*(*S* + 1) = 2

cutoff	spin error
0.01	1.5 × 10^–2^
0.005	8.7 × 10^–3^
0.001	9.3 × 10^–4^
0.0005	8.5 × 10^–4^
0.0001	2.0 × 10^–4^

## 4 Summary

We have demonstrated that 2-RDMs can be efficiently calculated to sufficient accuracy by using the compact wavefunctions of the selected configuration interaction approach of MCCI and by exploiting the structure of the wavefunction together with bitwise operations on modern CPUs. For this latter approach, we demonstrated that the calculation of the 2-RDM from a FCI wavefunction can be accelerated around 220 times, while a speed up of about 26 was shown for a MCCI wavefunction. This enabled us to investigate the accuracy of the 2-RDM when using truncated CI and MCCI for a set of systems that ranged from having almost no multireference character to being very strongly multireference. The general behavior of the errors for the 2-RDM compared with those for the energy or wavefunction was fairly similar.

Even for ground-state neon, which is well-described by methods based on small corrections to a single reference, we found that the stochastic selection of configurations in MCCI could produce wavefunctions that gave a lower error than CISDT for the 2-RDM, despite using fewer configurations than CISD. For the multireference system of excited neon, this improvement in accuracy was more pronounced where all the MCCI results had lower errors than CISD, and errors lower than CI(4) using fewer determinants than CISDT could be achieved.

For CO in its equilibrium geometry, which only has some multireference character, MCCI could reach a similar accuracy to CI(4), again only requiring roughly the same amount of determinants as CISDT. When the CO bond was stretched to 4 *a*_0_, the system was strongly multireference, and all the errors were noticeably larger than that for the equilibrium geometry. To make the truncated CI errors in the stretched geometry similar to CISD in the equilibrium geometry, we had to go to CI(4) for the energies but CI(5) for the 2-RDM. MCCI could achieve a 2-RDM accuracy similar to CI(6) but using fewer determinants than CI(4). When increasing the size of the basis set to cc-pVDZ for the equilibrium geometry, we found that, although MCCI had much higher accuracy than CISDT, it could not reach that of CI(4) due to the larger FCI space. CI(4) used around 2.8 million determinants, and the multireference character is not strong here. For this singlet system, we also considered the use of CSFs for the 2-RDM calculation and found that the main benefit seemed to be a reduced size of the wavefunction in the CSF representation as the spin contamination for MCCI with SDs was not large. Finally, we looked at CSFs when constructing the 2-RDM for the *M*_s_ = 1 triplet state of oxygen. Now, the reduction in the size of the wavefunction when using CSFs was not observed, unlike for the CO singlet. However, again, the spin contamination was very low when SDs were used.

The efficient calculation of the 2-RDM enabled these comparisons with FCI 2-RDMs calculated from wavefunctions as large as 2,414,950,976  determinants. These large FCI 2-RDMs should provide useful benchmark data for developers of 2-RDM functional theory.^57^ Previous work has demonstrated that the selected CI approach of MCCI can construct wavefunctions that capture the energy^58,59^ or multipoles^60^ accurately, yet use only a very small fraction of the FCI space. Now, we have shown that for the considered systems, the FCI 2-RDM can also be efficiently computed with sufficient accuracy using MCCI. This is particularly true for systems with strong multireference character and can be achieved with significantly fewer configurations than with truncated CI. Therefore, this work paves the way for the efficient calculation of any properties of multireference systems that depend on the 2-RDM, including analytic energy gradients and X-ray scattering.
